# Demographic, psychological, chronobiological, and work-related predictors of sleep disturbances during the COVID-19 lockdown in Italy

**DOI:** 10.1038/s41598-021-90993-y

**Published:** 2021-06-01

**Authors:** Federico Salfi, Marco Lauriola, Aurora D’Atri, Giulia Amicucci, Lorenzo Viselli, Daniela Tempesta, Michele Ferrara

**Affiliations:** 1 Department of Biotechnological and Applied Clinical Sciences, University of L’Aquila, Via Vetoio (Coppito 2), 67100 Coppito, AQ Italy; 2 Department of Social and Developmental Psychology, Sapienza University of Rome, Rome, Italy; 3 Department of Psychology, Sapienza University of Rome, Rome, Italy

**Keywords:** Circadian rhythms and sleep, Stress and resilience, Psychology

## Abstract

The first COVID-19 contagion wave caused unprecedented restraining measures worldwide. In Italy, a period of generalized lockdown involving home confinement of the entire population was imposed for almost two months (9 March–3 May 2020). The present is the most extensive investigation aimed to unravel the demographic, psychological, chronobiological, and work-related predictors of sleep disturbances throughout the pandemic emergency. A total of 13,989 Italians completed a web-based survey during the confinement period (25 March–3 May). We collected demographic and lockdown-related work changes information, and we evaluated sleep quality, insomnia and depression symptoms, chronotype, perceived stress, and anxiety using validated questionnaires. The majority of the respondents reported a negative impact of confinement on their sleep and a delayed sleep phase. We highlighted an alarming prevalence of sleep disturbances during the lockdown. Main predictors of sleep disturbances identified by regression models were: female gender, advanced age, being a healthcare worker, living in southern Italy, confinement duration, and a higher level of depression, stress, and anxiety. The evening chronotype emerged as a vulnerability factor, while morning-type individuals showed a lower predisposition to sleep and psychological problems. Finally, working from home was associated with less severe sleep disturbances. Besides confirming the role of specific demographic and psychological factors in developing sleep disorders during the COVID-19 pandemic, we propose that circadian typologies could react differently to a particular period of reduced social jetlag. Moreover, our results suggest that working from home could play a protective role against the development of sleep disturbances during the current pandemic emergency.

## Introduction

The rapid spread of the new Coronavirus (SARS-CoV-2) outbreak led the global governments to adopt generalized lockdown and social distancing measures to limit the virus propagation. The Italian population was subjected to home confinement for almost two months (9 March–3 May 2020). The restraining measures involved unprecedented limitations of mobility rights and social interactions. This stressful situation profoundly compromised the general population's everyday life, resulting in pervasive psychological repercussions^1,2^. In this extraordinary historical period, sleep could represent one of the primary victims^3^. Several aspects could contribute to sleep disturbances during this period. The fear of contagion, the uncertainty of the future, the low activity levels during the day, and the reduction of social interactions could all be factors associated with developing sleep problems. Notably, the home confinement disrupted the daily activity routine, compromising the daylight exposure, which is a crucial regulator of the circadian rhythms. Several cross-sectional and longitudinal studies confirmed this assumption, showing a high prevalence of sleep disturbances during the lockdown period (for a meta-analysis^4^). Meanwhile, there was a worldwide increase in electronic backlit screen exposure, which was suggested as a contributing factor to sleep deterioration in the long run^5^.

In the current study, we present the most extensive investigation (N = 13,989) aimed to provide a comprehensive snapshot of sleep health and habits during the entire home confinement period due to the COVID-19 outbreak. We used a web-based survey covering the two months of total lockdown to evaluate sleep quality (by means of the Pittsburgh Sleep Quality Index—PSQI^6,7^), insomnia symptoms (Insomnia Severity Index—ISI^8,9^), chronotype (reduced version of the Morningness-Eveningness Questionnaire—MEQr^10^), depression symptoms (Beck Depression Inventory-second edition—BDI-II^11^), perceived stress (10-item Perceived Stress Scale—PSS-10^12^), and anxiety (state-anxiety subscale of the State-Trait Anxiety Inventory—STAI-X1^13^) of the Italian population under restraining measures. The present investigation was conceived to understand the sociodemographic, psychological, chronobiological, and work-related predictors of the sleep disturbances during the home confinement period.

Several cross-sectional studies showed women^14–18^ and healthcare workers^19,20^ as the categories suffering the most. Furthermore, literature also revealed a close relationship between psychological well-being and sleep disturbances during the pandemic period^4,16,21,22^. We first of all expected to confirm these results within our larger sample.

Moreover, the present study aimed to provide new insights about some aspects not yet addressed, such as the role of the individual circadian preference (chronotype) and the lockdown-related work changes in the expression of sleep disturbances during the first COVID-19 outbreak wave.

The restraining measures impacted the sleep/wake rhythms. People delayed the bedtime and wake-up time during the lockdown^18,21,23,24^, resulting in a reduction of social jetlag^23–25^, which could be conceived as a proxy for a challenged circadian system and compromised sleep^26,27^. Conventionally, three main chronotypes have been identified^28^: the morning-type (MT), the neither-type (NT), and the evening-type (ET). The ET typically showed lower sleep quality^29–31^, and this evidence was ascribed to the misalignment between the biological and social clock (i.e., the timing of social obligations), which is associated to the unhealthy manifestation of social jetlag^26,28^. Furthermore, higher levels of negative mood and anxiety characterize ET individuals^31–34^. The social jetlag and the sleep difficulties have been proposed to account for the ET's tendency to experience psychological symptoms^35–38^. In this view, we could expect that during a period of large-scale reduction of the social jetlag such as the home confinement due to the COVID-19 outbreak, the gap between circadian typologies could be narrowed down because ET, in particular, may have benefitted from a period marked by a loosening of rigorous sleep/wake schedule due to weaker social and working obligations.

Finally, the lockdown impacted the working routine of the majority of the population, leading to the suspension of the working activity and the imposition of remote working. The widespread possibility of working from home in telematic mode with a great flexibility in the working schedule could be a further factor contributing to the general population's sleep quality. Therefore, we aimed at verifying the influence of changes in working activity caused by the restraining measures on sleep habits.

## Results

### Lockdown-related consequences on sleep and prevalence of sleep disturbances

The majority of respondents reported a negative impact of the restraining measures on their sleep, delayed bedtime and wake-up time, and maintained unchanged nap habits. According to the PSQI and ISI criteria, over six out of ten of the participants were poor sleepers, and 15% of the respondents had symptoms of moderate/severe clinical insomnia (Table 1).

**Table 1:** Prevalence of lockdown-related consequences and sleep disturbance prevalence within the total sample.

	N (%)
**Perceived impact**	
Negative	8455 (60.5)
None	3306 (23.6)
Positive	2228 (15.9)
**Bedtime**	
Advanced	1288 (9.2)
Unchanged	4483 (32.1)
Delayed	8218 (58.7)
**Wake-up time**	
Advanced	1570 (11.2)
Unchanged	3563 (25.5)
Delayed	8856 (63.3)
**Nap habits**	
Increased	2477 (17.7)
Unchanged	9045 (64.7)
Reduced	2467 (17.6)
**Sleep quality (PSQI)**	
Poor	8053 (61.1)
Good	5122 (38.9)
**Insomnia (ISI)**	
No	6597 (47.2)
Subthreshold	5297 (37.9)
Moderate	1840 (13.2)
Severe	255 (1.8)

### Predictors of sleep disturbances

Significant regression equations were found with PSQI and ISI scores as dependent variables (PSQI: *R*^2^ = 0.30, *F*_17,8552_ = 219.07, *p* < 0.001; ISI: *R*^2^ = 0.35, *F*_17,9046_ = 285.07, *p* < 0.001).

As shown in Table 2, older age, female gender, healthcare work, living in southern Italy, and confinement duration were associated with highest PSQI and ISI scores. Moreover, lower education predicted poorer sleep quality. Lower scores of MEQr (pointing to evening chronotype), and a higher level of depression, perceived stress, and anxiety predicted poorer sleep quality and more severe insomnia symptoms.

**Table 2:** Results (*β* and *p*) of the multiple linear regressions on PSQI and ISI scores.

Predictor	PSQI score		ISI score	
	*β*	*p*	*β*	*p*
Intercept		< 0.001		0.129
Age	0.136	< 0.001	0.048	< 0.001
**Gender**				
Woman	*Reference*		*Reference*	
Man	− 0.182	< 0.001	− 0.087	< 0.001
**Education**				
Middle school	*Reference*		*Reference*	
High school	− 0.165	0.003	0.003	0.957
Graduated	− 0.218	< 0.001	− 0.063	0.224
Over graduated	− 0.256	< 0.001	− 0.082	0.151
**Occupation**				
Health work	*Reference*		*Reference*	
Other work	− 0.134	< 0.001	− 0.078	0.038
Student	− 0.201	< 0.001	− 0.142	< 0.001
Unemployed	− 0.084	0.088	− 0.038	0.404
**Geographic location**				
Northern Italy	*Reference*		*Reference*	
Central Italy	0.003	0.903	− 0.015	0.487
Southern Italy	0.094	< 0.001	0.040	0.054
Home confinement duration	0.073	< 0.001	0.068	< 0.001
**Forced quarantine**				
Yes	*Reference*		*Reference*	
No	− 0.053	0.142	− 0.006	0.859
No response	0.126	0.386	0.230	0.097
MEQr score	− 0.079	< 0.001	− 0.070	< 0.001
BDI-II score	0.330	< 0.001	0.369	< 0.001
PSS-10 score	0.073	< 0.001	0.097	< 0.001
STAI-X1 score	0.148	< 0.001	0.157	< 0.001

*MEQr* Morningness-Eveningness Questionnaire-reduced, *BDI-II* Beck Depression Inventory-second edition, *PSS-10* Perceived Stress Scale-10 item, *STAI-X1* state-anxiety subscale of the State-Trait Anxiety Inventory.

^a^Northern Italy: Aosta Valley, Emilia Romagna, Friuli-Venezia Giulia, Liguria, Lombardy, Piedmont, Trentino-Alto Adige, and Veneto.

^b^Central Italy: Lazio, Marche, Tuscany, and Umbria.

^c^Southern Italy: Abruzzo, Apulia, Basilicata, Calabria, Campania, Molise, Sardinia, and Sicily.

### Chronotype differences

According to the MEQr criteria, our sample consisted of 3261 MT subjects (21.3%), 9181 NT (65.6%), and 1547 ET (11.1%). Chi-square tests (Table 3) showed significant associations of the chronotype group (MT, NT, ET) with the perceived impact of the restraining measures, with the reported changes in bedtime, wake-up time, and nap frequency, and with the prevalence of poor sleepers and clinical insomnia conditions**.** In particular, a higher proportion of ET subjects reported a negative impact of the restraining measures, delayed bedtime and wake up time, and changes of nap habits. Additionally, the ET group was marked by a higher prevalence of poor sleepers and clinical insomniacs. MT participants showed the opposite pattern of results.

**Table 3:** Frequency of the lockdown-related perceived impact on sleep, reported changes of bedtime, wake-up time and nap habits, and prevalence of poor sleepers and clinical insomnia conditions within the three chronotype groups (MT, NT, ET).

		N (%)			*χ*^*2*^	*p*
		MT	NT	ET		
Perceived impact	Negative	1645 (50.4)	5740 (62.5)	1067 (69.0)	211.02	< 0.001
	None	1017 (31.2)	2015 (21.9)	277 (17.9)		
	Positive	599 (18.4)	1426 (15.5)	203 (13.1)		
Bedtime	Advanced	384 (11.8)	820 (8.9)	84 (5.4)	480.29	< 0.001
	Unchanged	1433 (43.9)	2752 (30.0)	298 (19.3)		
	Delayed	1444 (44.3)	5609 (61.1)	1165 (75.3)		
Wake-up time	Advanced	454 (13.9)	994 (10.8)	122 (7.9)	419.39	< 0.001
	Unchanged	1184 (36.3)	2146 (23.4)	233 (15.1)		
	Delayed	1623 (49.8)	6041 (65.8)	1192 (77.1)		
Nap habits	Increased	563 (17.3)	1575 (17.2)	339 (21.9)	59.84	< 0.001
	Unchanged	2223 (68.2)	5926 (64.5)	896 (57.9)		
	Reduced	475 (14.6)	1680 (18.3)	312 (20.2)		
Sleep quality	Poor	1599 (51.9)	5433 (62.6)	1021 (72.2)	190.25	< 0.001
	Good	1481 (48.1)	3247 (37.4)	394 (27.8)		
Insomnia	No	1901 (58.3)	4131 (45.0)	565 (36.5)	291.11	< 0.001
	Subthreshold	1041 (31.9)	3614 (39.4)	642 (41.5)		
	Moderate	288 (8.8)	1264 (13.8)	288 (18.6)		
	Severe	31 (1.0)	172 (1.9)	52 (3.4)		

Chi-square test results are also reported (*χ*^2^ and *p*).

*MT* Morning-type, *NT* Neither-type, *ET* Evening-type.

**Figure 1:**
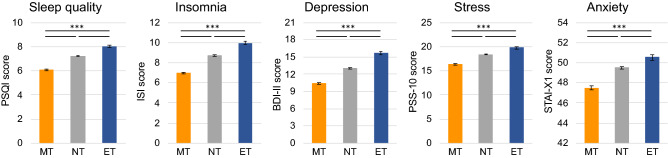
Sleep quality (PSQI), insomnia severity symptoms (ISI), depression (BDI-II), perceived stress (PSS-10), and anxiety (STAI-X1) for the three chronotype groups (Morning-type—MT, Neither-type—NT, Evening-type—ET). Means (and standard errors) of questionnaire scores are reported. Results of Bonferroni post hoc comparisons are reported with asterisks (***p < 0.001).

As hypothesized, the MEQr scores and the age variable were highly correlated (*R* = 0.28; *p* < 0.001), confirming the assumption of using the age as covariate in the subsequent analyses. One-way ANCOVAs carried out on sleep and psychological questionnaires showed a significant effect of the “chronotype” factor (MT, NT, ET) (PSQI: *F*_13171_ = 152.70, *p* < 0.001; ISI: *F*_13985_ = 173.01, *p* < 0.001; BDI-II: *F*_9978_ = 134.17, *p* < 0.001; PSS-10: *F*_9278_ = 95.01, *p* < 0.001; STAI-X1: *F*_9060_ = 45.58, *p* < 0.001). The “age” covariate yielded a significant effect in the analyses of PSQI, BDI-II, and PSS-10 scores (all *p* < 0.001), while it was not significant for ISI (*p* = 0.35) and STAI-X1 (*p* = 0.58). Post hoc comparisons (Fig. 1) showed that the ET group had higher scores in all the dimensions compared to MT and NT (all *p* < 0.001). NT showed higher scores compared to MT group for all the variables (all *p* < 0.001).

Control analyses were performed adding the “gender” factor as a further covariate in the ANCOVAs, confirming all the above-reported pattern of results. Finally, exploratory analyses highlighted a significant difference between the three chronotype groups for the reported sleep duration (*F*_13986_ = 14.14, *p* < 0.001). ET participants slept more (mean ± SEM, 444 min ± 2 min) than NT (426 min ± 1 min) and MT groups (420 min ± 1 min; both *p* < 0.001).

### Working activity changes

A total of 3314 workers (38.9% of the total workers' sample) suspended their working activity during the lockdown. *T*-tests on PSQI and ISI scores showed significant differences between the group of respondents that suspended and the group that preserved their working activity (PSQI: *t*_8084_ = 2.56, *p* = 0.01; ISI: *t*_8523_ = 6.18, *p* < 0.001). The suspension of the working activity was associated with lower sleep quality (mean ± SEM, 7.15 ± 0.07 vs. 6.91 ± 0.05) and more severe insomnia symptoms (8.67 ± 0.10 vs. 7.91 ± 0.08).

Among the sample who continued to work (5211 subjects), a total of 3536 respondents worked from home, 2125 reported reduced working time, 1989 maintained unchanged the work duration, and 1097 subjects increased their daily working time.

Two-way ANOVAs on PSQI and ISI scores highlighted significant effects of “remote working” (PSQI: *F*_1,4941_ = 45.91, *p* < 0.001; ISI: *F*_1,5205_ = 17.60, *p* < 0.001), and “daily working time” factors (PSQI: *F*_2,4941_ = 28.49, *p* < 0.001; ISI: *F*_2,5205_ = 25.21, *p* < 0.001). The interactions between the two factors (“remote working” x “daily working time”) were significant in both analyses (PSQI: *F*_2,4941_ = 6.23, *p* = 0.002; ISI: *F*_2,5205_ = 4.13, *p* = 0.02).

**Figure 2:**
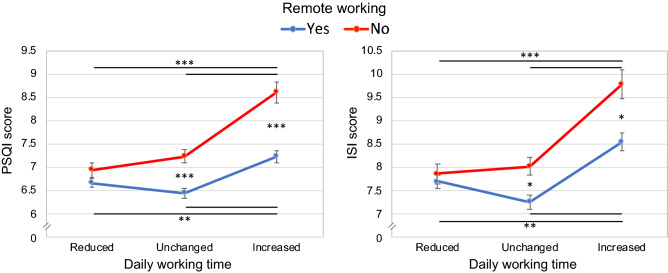
Interaction between "remote working" (yes, no) and "daily working time" (reduced, unchanged, increased) factors, for sleep quality (PSQI) and insomnia severity symptoms scores (ISI). The figures report means (and standard errors) of the scores of the PSQI and ISI questionnaires. Results of Bonferroni post hoc comparisons are reported with asterisks (*p < 0.05; **p < 0.01 ***p < 0.001).

Post hoc comparisons (Fig. 2) pointed to lower sleep quality and more severe insomnia symptoms for the participants who increased the daily working time within both remote working and regular working group (all *p* < 0.01). There were no differences in PSQI and ISI scores between the two groups (remote vs. regular work) when they reduced the working time (both *p* = 1.00). When the daily working time was the same or increased compared with the pre-outbreak period, the remote workers showed lower scores on PSQI (both *p* < 0.001) and ISI questionnaires (*p* = 0.03, *p* = 0.01; respectively). Notably, the remote workers who increased the daily working time reported the same sleep quality and insomnia symptoms of the regular workers who reduced (PSQI: *p* = 1.00; ISI: *p* = 0.25) or maintained unchanged the working duration (PSQI: *p* = 1.00; ISI: *p* = 0.85).

Exploratory analyses showed that the remote working group went to bed and woke-up later (bedtime: hh:mm ± SEM, 00:01 ± 1 min; wake-up time: 08:17 ± 1 min) and slept more (419 min ± 1 min) compared to the respondents who continued to reach the workplace (bedtime: 23:33 ± 2 min, *t*_5209_ = 11.93, *p* < 0.001; wake-up time: 07:28 ± 2 min, *t*_5209_ = 19.20, *p* < 0.001; sleep duration: 392 min ± 2 min, *t*_5209_ = 11.38, *p* < 0.001).

## Discussion

Our study provided a comprehensive description of sleep health during the lockdown in Italy. The majority of respondents (approximately 60%) declared a negative impact of the restraining measure and delayed bedtime and wake-up time. We highlighted an alarming prevalence of poor sleepers and clinical insomniacs within our large sample: six out of ten participants were poor sleepers and more than half of the sample presented from subthreshold to severe insomnia symptoms.

On the other hand, it is noteworthy that a proportion of the respondents (approximately 16%) declared a positive impact of the lockdown measures on their sleep, supporting the assumption that a loosening of rigorous sleep/wake schedule due to weaker social and working obligations could have a beneficial effect on part of the population^39^.

In line with the current literature^4,16,21,22^, we demonstrated a strong relationship between sleep variables and depression, stress, and anxiety. We confirmed the results of other cross-sectional investigations on healthcare workers^19,20^ and women^14–18^, which appeared as the categories experiencing the most severe sleep problems during lockdown worldwide. Furthermore, advanced age predicted more severe sleep disturbances. An interpretation of the healthcare workers' results is related to the well-known increased stressful workload, accompanied by higher contagion risk. Consistently, several studies demonstrated a high prevalence of post-traumatic stress disorder (PTSD) symptoms and mental health problems among the healthcare operators during the COVID-19 pandemic^20,40^. On the other hand, we suggest caution in the interpretation of the results on women and elderly population since these two factors were typically associated with the poorest sleep quality^41,42^ and the higher predisposition to insomnia conditions even in the pre-outbreak period^43,44^. Consistently, our recent longitudinal study^45^ showed that the time course of sleep disturbance was different between men and women, and the male gender appeared as the most vulnerable to the prolongation of the restraining measures.

We highlighted more severe sleep disturbances in southern Italy, and this result is inconsistent with the available literature on sleep and COVID-19 in the Italian population^17^, which identified a higher prevalence of sleep problems in northern Italy. However, differences in the period under consideration might explain the different results. The previous investigation^17^ was referred to the first weeks of the outbreak, when the geographic propagation of the contagion in Italy was extremely unbalanced towards northern regions^46^. Our study covered the entire confinement period instead, providing a more reliable overview of the effect of the pandemic propagation in southern Italy. Moreover, we hypothesize that our results could also reflect the pandemic's economic consequences since southern Italy was the territorial area most affected economically by the COVID-19 crisis^47^. Finally, the confinement duration was a predictive factor of sleep disturbances, corroborating the hypothesis of a cumulative detrimental effect of the protracted lockdown period. However, scarce evidence had been provided worldwide, with only a few studies addressing this question through longitudinal investigations across the confinement period^5,45,48^.

### Chronotypes under lockdown

As far as the influence of chronotypes is concerned, according to the initial hypothesis, ET respondents reported the most prevalent delay of the sleep phase. These results are consistent with another Italian cross-sectional study carried out during the lockdown^49^. However, the ET participants reported suffering more from the confinement situation than the other circadian typologies. Coherently, this group showed the lowest sleep quality and the highest level of insomnia, depression, perceived stress, and anxiety. Meanwhile, MT showed the opposite pattern of results, declaring a lower negative impact of the restraining measures and a higher prevalence of preserved sleep schedule. This finding was exemplified by the highest sleep quality, less marked insomnia and depression symptoms, and the lowest perceived stress and anxiety levels.

The present results pointed to a particular vulnerability of the ET group, although the lockdown was a favourable period to reduce the mismatch between internal and social clocks. Of note, the present results are consistent with those obtained during the pandemic period in an adolescent clinical population^50^.

Our findings suggest that the well-known higher predisposition to sleep disturbances of the ET people^29–31^ should not be considered only as a consequence of the accumulated sleep debt due to social and working obligations. In fact, during an unprecedented condition that unlocked time for sleep^27^, the ET respondents paradoxically reported a slighty longer sleep time althought they typically slept less than other circadian typologies in the workdays of pre-pandemic period^28^, however preserving the more severe sleep problems. This evidence suggests that the evening-individuals' sleep disturbances may instead originate from the misalignment of the delayed sleep pattern to the biological night^51^, which became more pronounced during the lockdown.

On the other hand, morning chronotype emerged as a protective factor, both on the sleep and psychological sides. Recent studies demonstrated that the three chronotype groups differ for resilience level^52–54^ and perceived stress^55^. The morning chronotype seems to be more able to cope with challenging situations, while ET individuals are more predisposed to develop PTSD^56,57^. Our findings confirm this assumption, even in a context of reduced social jetlag, such as during the lockdown.

Finally, several studies showed that circadian typologies are associated with particular personality traits and social behavior^58,59^, which could interact with the period of restraining measures^60^, contributing in explaining our pattern of results.

### Working during pandemic

As expected, a substantial percentage of participants suspended the working activity during the lockdown (38.9%), leading to a lower sleep quality and more severe insomnia symptoms. We believe that these findings can be ascribable to the economic repercussions of the work interruption, although this dimension was not assessed in the present study. However, the possibility of maintaining a regular working activity during the confinement could have had a direct positive impact on preserving sleep health. The absence of a daily activity routine could emphasize the sense of boredom, leading to a slowing of the felt pace of the time flow^61^. Consistently, a recent study demonstrated a relationship between the increase of sleep difficulties and the feeling of time dilatation during the lockdown period^21^. Coherently, in our study, the unemployed participants were the only group that did not differ from the healthcare workers for sleep disturbances.

On the other hand, within the group of respondents that continued to work, remote working seemed to be a protective factor. During this particular historical period, working from home was strictly associated with a reduced likelihood of contagion, and thus to a lower perception of risk. Moreover, the higher flexibility of the working schedule could encourage a better organization of the sleep/wake rhythms, favouring longer sleep duration. Consistently, the remote workers slept almost half an hour more than those who continued to reach the workplace.

The changes in daily working time emerged as an essential predictor of sleep disturbances, and the increased work hours were associated with significant sleep problems. This result is consistent with other studies showing an adverse effect of the increased work routine on sleep quality and quantity^62,63^. Interestingly, when working time was reduced, there was no benefit of remote working. On the other hand, when the working schedule was maintained or increased, participants who worked remotely showed better sleep quality and fewer insomnia symptoms. However, when the remote workers increased their daily working time, they reached the sleep disturbance level of the regular workers who maintained/reduced the working hours. Therefore, working remotely during the current pandemic should be encouraged as a protective factor, focusing on avoiding extra working time. When the regular working day is not punctuated by fixed starting and ending time point, a common consequence could be the increase of daily working duration, with negative consequences on sleep.

## Conclusions

To the best of our knowledge, the present is the most extensive investigation aimed at understanding the pandemic-related consequences on the general population's sleep. However, it should be acknowledged that we used a non-probabilistic sampling technique, with a higher representativity of the female gender and the young population, and the information was collected via self-report questionnaires. Moreover, no data referred to the pre-pandemic period are available, and the cross-sectional nature of the present study precludes causal conclusions regarding the relationship between the examined dimensions.

The results confirmed the hypothesis that the lockdown due to the COVID-19 outbreak had significant repercussions on the sleep quality of the general population^3^.

The restraining measures had a cumulative cost, and our results confirm the need to avoid over precautionary approaches, keeping the home confinement period as short as possible to limit its long-term negative consequences for sleep and mental health^64^.

Our results are consistent with the current literature suggesting a higher predisposition of the female gender to develop sleep problems. In addition, the healthcare workers emerged again as an at-risk category. Moreover, our results showed that the differences in individual daily activity pattern preferences could represent a crucial predictor of sleep and psychological health during the pandemic period. We demonstrated a particular vulnerability of the ET people, while the morning chronotype seems to be a protective factor during the current challenging period.

In light of this evidence, the vulnerable categories should be placed at the center of preventive interventions to avoid the exacerbation of sleep disturbances and mental health problems in the long run. Chronobiological interventions, such as melatonin, light exposure, and social rhythm regulation, could be effective strategies for ET people to hinder the onset or exacerbation of depression symptoms during the period of restraining measures^28^.

In conclusion, we propose some guidelines for working during the COVID-19 pandemic. Individuals who suspended the working activity should maintain a regular daily activity to counteract the development and exacerbation of sleep disturbances. Remote working should be encouraged, as long as the overall daily activity duration does not increase, establishing fixed starting and ending times of the workday. This aspect should be regulated since remote working and teleworking could become increasingly widespread modalities regardless of the pandemic's conclusion^65^. In this view, the results of the present investigation could be generalizable to non-emergency periods. Furthermore, the subjects who work in telematic modality should avoid exposure to backlit screens of electronic devices before falling asleep since the increased evening exposure was suggested as a causal factor in developing sleep disturbances during lockdown^5^.

An adequate sleep quality/quantity is essential to deal with stressful events^66^ and preserve mental health^67^, emotional regulation^68,69^, as well as the proper functioning of the immune system^70^. Consequently, the present results have a broad-spectrum of implications.

Our study's findings could be essential in the present period, where the second contagion wave has become a reality, hundreds of thousands of people are subjected to restraining measures worldwide, and the impact of current emergency on sleep and mental health of general population persists^71^.

All the insights provided in this study should be considered from the institutions to design public campaigns aimed to promote sleep health and general well-being during the current unprecedented situation.

## Methods

### Participants and experimental procedure

A web-based survey has been disseminated through social media (Facebook, WhatsApp, Instagram, Twitter) from the third week to the end of the confinement period (25 March–3 May 2020), using a snowball sampling technique. A total of 13,989 Italian citizens (mean age ± standard deviation, 34.8 ± 12.2 years, range 18–86, 3223 males) participated in the present investigation. The survey started with demographic questions (age, gender, education, occupation, geographic location) and COVID-related information (infection or forced quarantine). Demographic informations are reported in Table 4. Then, we asked to rate the perceived impact of the lockdown on sleep quality (positive, none, negative), and the occurred changes of bedtime (delayed, maintained, advanced), wake-up time (delayed, unchanged, advanced), and nap habits (increased, unchanged, reduced). Then, the survey comprised an evaluation of the working activity changes. In particular, we collected information on the suspension of the working activity (yes, no), the beginning of the remote working modality (yes, no), and the changes of the daily working time (increased, unchanged, reduced). Subsequently, we evaluated sleep quality, insomnia severity symptoms, and chronotype, through a set of validated questionnaires (see next paragraph for a detailed description): the Pittsburgh Sleep Quality Index (PSQI^6,7^), the Insomnia Severity Index (ISI^8,9^), the reduced version of the Morningness-Eveningness Questionnaire (MEQr^10^). Finally, we assessed depression symptoms, perceived stress, and anxiety using (in order of presentation) the Beck Depression Inventory-second edition (BDI-II^11^), the 10-item Perceived Stress Scale (PSS-10^12^), and the state-anxiety subscale of the State-Trait Anxiety Inventory (STAI-X1^13^), respectively. Participation in the entire survey required approximately 25 min, and the compilation of the last three questionnaires (BDI-II, 10-PSS, STAI-X1) was optional to avoid false/unreliable responses in the final part of the survey. A total of 9982 respondents (71.4%) compiled the BDI-II, 9282 also the 10-PSS (66.5%), and 9064 completed all the questionnaires (64.8%). The study was approved by the institutional review board of the University of L’Aquila (protocol n. 43066/2020) and carried out according to the principles established by the Declaration of Helsinki. Online informed consent to participate in the research was obtained from all the respondents.

**Table 4:** Demographic characteristics of the sample.

	N (%)
**Age**	
18–30	7424 (53.0)
31–50	4755 (33.9)
Over 50	1810 (12.9)
**Gender**	
Men	3123 (22.3)
Women	10,866 (77.6)
**Geographical location**	
Northern Italy^a^	5783 (41.3)
Central Italy^b^	3389 (24.2)
Southern Italy^c^	4817 (34.4)
**Education**	
Middle school	501 (3.6)
High school	5350 (38.2)
Graduated	6750 (48.2)
Over graduated	1388 (9.9)
**Occupation**	
Unemployed	1347 (9.6)
Student	4117 (29.4)
Worker	
Healthcare work	781 (5.6)
Other work	7744 (55.3)
**COVID-19 infection**	
No	13,801 (98.6)
Yes	44 (0.3)
No response	144 (1.0)
**Forced quarantine**	
No	12,890 (92.1)
Yes	1032 (7.4)
No response	67 (0.5)

^a^Northern Italy: Aosta Valley, Emilia Romagna, Friuli-Venezia Giulia, Liguria, Lombardy, Piedmont, Trentino-Alto Adige, and Veneto.

^b^Central Italy: Lazio, Marche, Tuscany, and Umbria.

^c^Southern Italy: Abruzzo, Apulia, Basilicata, Calabria, Campania, Molise, Sardinia, and Sicily.

### Questionnaires

The PSQI is a 19-item questionnaire widely used to evaluate sleep quality^6,7^. Each dimension covered by PSQI (sleep quality, sleep duration, sleep latency, habitual sleep efficiency, sleep disorders, the use of sleeping medications, daytime dysfunctions) is scored between 0 and 3, and higher scores (range, 0–21) point to more severe sleep difficulties. Scores higher than 5 represent a valid indicator of poor sleep quality^6^.

The ISI is a screening instrument to assess the severity of clinical insomnia^8,9^. It comprises an evaluation of seven dimensions: difficulty falling asleep, difficulty staying asleep, waking too early, sleep satisfaction, sleep interference with daytime functioning, noticeability of sleep problems by others, and worry about sleep. Respondents rate each item using a 5-point Likert scale (0–4), yelding a total score ranging from 0 to 28. Validated cut-off scores can be used to identify clinical insomnia conditions (0–7: no insomnia; 8–14 subthreshold insomnia; 15–21: moderate insomnia; 22–28: severe insomnia)^8^.

The MEQr is a 5-item questionnaire comprising a self-report evaluation of ideal rising time and bedtime, personal efficiency peak time, morning freshness, and self-evaluation of chronotype^10^. It represents a short version of the original 19-item mixed-format scale developed by Horne and Östberg^72^, which is the most widely used self-report instrument in chronopsychological research to identify circadian typologies^28^. Discriminating power of the Italian version of MEQr to identify circadian typologies was confirmed using physiological measures (body temperature) and recorded motor activity as external criterion^10,73^. Total score ranging from 4 to 25 is used to classify the chronotype (ET: 4–10; NT: 11–18; MT: 19–25).

The BDI-II is a 21-item self-report inventory designed to measure the severity of depression assessing affective, somatic, and cognitive symptoms according to diagnostic criteria listed in the Diagnostic and Statistical Manual for Mental Disorders^74^. Respondents rate the severity of each symptom using a 0 to 3 scale, and higher scores indicate more severe depression symptomatology (range, 0–63).

The PSS-10 is a reduced version of the widely used PSS^75^. It is a 10-item questionnaire evaluating thoughts and feelings referred to stressful events. Respondents are asked how often they felt a certain way on a 0–4 Likert scale regarding six negatively stated and four positively stated (reverse score) items. Higher scores point to higher perceived stress (range, 0–40). The Italian version of PSS-10 showed greater psychometric properties than the original PSS^12^.

The STAI-X1^13^ is a well-established instrument to measure state anxiety in research and clinical settings. It is a subscale of the State-Trait Anxiety Inventory, included in the Cognitive Behavioural Assessment battery 2.0^76^. STAI-X1 comprises 20 item items referred to feelings of of apprehension, tension, nervousness, worry, and activation of the autonomic nervous system. Respondents rate the intensity of each symptom on a 4-point Likert scale; higher scores indicate more significant state anxiety (range, 20–80).

### Data analysis

We performed frequencies analyses to show the proportion of the reported impact of the lockdown period on sleep (negative, none, positive), and the changes of bedtime (advanced, unchanged, delayed), wake-up time (advanced, unchanged, delayed), and nap habits (increased, unchanged, reduced).

The analyses involving PSQI score were carried out excluding 814 participants due to compilation errors (i.e., respondents declared longer total sleep time compared with the reported total time in bed). According to the validated criteria of PSQI and ISI questionnaires, we calculated the prevalence of poor sleepers and clinical insomniacs in order to provide a descriptive overview of the entire sample.

Multiple regression analyses were carried out with PSQI and ISI scores as dependent variables. The regression models comprised the following continuous and categorical predictors: age (continuous variable), gender (man, woman), education (middle school, high school, graduated, over-graduated), occupation (healthcare work, other work, student, unemployed), geographic location (norther Italy, central Italy, southern Italy), the experience of the forced quarantine (yes, no, no response), duration of the confinement period (based on the day of participation to the survey), and the MEQr, BDI-II, PSS-10, and STAI-X1 questionnaire scores (continuous variables). We did not include the COVID-19 infection factor (yes, no, no response) due to the low number of infected subjects (only 44 participants).

We calculated the chronotype composition of our sample (MT, NT, ET) according to the MEQr cut-off scores. Then, we performed chi-square tests to evaluate the association of the three circadian typology groups with the perceived impact of the lockdown period on sleep, the reported changes of bedtime, wake-up time, and nap habits, and the prevalence of poor sleepers and clinical insomnia conditions.

To evaluate differences in sleep quality, insomnia severity symptoms, depression, perceived stress, and anxiety, between MT, NT, and ET, we carried out ANCOVAs on the scores of the five questionnaires (PSQI, ISI, BDI-II, PSS-10, STAI-X1), with chronotype (MT, NT, ET) as three-level between factor. The current literature supports a strong relationship between age and chronotype^28,77^. Therefore, the analyses controlled for the effect of age (continuous variables) used as a covariate.

Finally, we applied a *t*-test analysis to compare the PSQI and ISI scores of the respondents who suspended or maintained the working activity. In order to evaluate the effect on sleep quality and insomnia symptoms due to the changes in the modality (remote working) and duration of the daily working activity, the PSQI and ISI scores were submitted to two-way ANOVAs, with "remote working" (yes, no) and "daily working time" (increased, unchanged, reduced) as two-level and three-level between factors, respectively.

In some cases, further exploratory analyses were performed, using the information of interest (i.e., bedtime, wake-up time, sleep duration) derived from the PSQI (see "Results" paragraph).

All the analyses were two-tailed, and Bonferroni post hoc comparisons were performed in case of significant effects. *p*-value < 0.05 was considered significant. When the data did not appear normally distributed or looked like heteroscedastic, "robust" or nonparametric techniques were used to check for bias in the inferential tests that could have led to misleading conclusions. Because these control analyses produced almost identical results to those obtained using the standard parametric tests, we concluded that violations of parametric assumptions were of negligible importance and we reported only the parametric test results.

## Data Availability

The datasets analysed during the current study are available from the corresponding author on reasonable request.
